# Strength in Diversity: Nuclear Export of Viral RNAs

**DOI:** 10.3390/v12091014

**Published:** 2020-09-11

**Authors:** Jón Pol Gales, Julie Kubina, Angèle Geldreich, Maria Dimitrova

**Affiliations:** 1 Institut de Biologie Moléculaire des Plantes, The French National Center for Scientific Research (CNRS) UPR2357, Université de Strasbourg, F-67084 Strasbourg, France; jpgales@etu.unistra.fr (J.P.G.); julie.kubina@inrae.fr (J.K.); angele.geldreich@ibmp-cnrs.unistra.fr (A.G.); 2 SVQV UMR-A 1131, INRAE, Université de Strasbourg, F-68000 Colmar, France

**Keywords:** virus, nucleus, viral RNA nuclear export, viral ribonucleoprotein complexes

## Abstract

The nuclear export of cellular mRNAs is a complex process that requires the orchestrated participation of many proteins that are recruited during the early steps of mRNA synthesis and processing. This strategy allows the cell to guarantee the conformity of the messengers accessing the cytoplasm and the translation machinery. Most transcripts are exported by the exportin dimer Nuclear RNA export factor 1 (NXF1)–NTF2-related export protein 1 (NXT1) and the transcription–export complex 1 (TREX1). Some mRNAs that do not possess all the common messenger characteristics use either variants of the NXF1–NXT1 pathway or CRM1, a different exportin. Viruses whose mRNAs are synthesized in the nucleus (retroviruses, the vast majority of DNA viruses, and influenza viruses) exploit both these cellular export pathways. Viral mRNAs hijack the cellular export machinery via complex secondary structures recognized by cellular export factors and/or viral adapter proteins. This way, the viral transcripts succeed in escaping the host surveillance system and are efficiently exported for translation, allowing the infectious cycle to proceed. This review gives an overview of the cellular mRNA nuclear export mechanisms and presents detailed insights into the most important strategies that viruses use to export the different forms of their RNAs from the nucleus to the cytoplasm.

## 1. Introduction

The hallmark difference between eukaryotic and prokaryotic life is the presence of the nucleus. Limited by the double lipidic bilayer nuclear envelope, the nucleus physically separates the genetic material from the cytoplasm, decoupling the transcriptional and translational steps of gene expression. The nucleus remains connected to the cytoplasm through the nuclear pore complex (NPC), the gatekeeper allowing the nucleocytoplasmic transport of selected macromolecules [1,2,3].

Viruses represent the most abundant and genetically diverse biological entities on Earth (reviewed in [4]) and infect virtually every organism. Interestingly, when comparing the known viromes of eukaryotes and prokaryotes, there is a notable difference in the abundance of the types of viral genomes [5]. In prokaryotes, the majority of viruses possess a double-stranded DNA genome, allowing them to easily exploit the host’s DNA replication and transcription machineries [5]. In eukaryotes, the situation is the opposite. The majority of viruses have a positive-strand RNA genome and are capable of replicating within the cytoplasm, negating the need for access to the nucleus [5]. DNA viruses are comparatively rare, which is likely at least partially due to the appearance of the nuclear envelope, which created new evolutionary challenges for viruses requiring access to the nucleus. Among these challenges is the initial nuclear import of the viral genome, the nuclear export of viral RNAs for translation, and the trafficking of viral proteins to and from the nucleus. Once these challenges are overcome, however, the nucleus represents a new evolutionary niche that some viruses have exploited with remarkable success. The most prominent example might be retroviruses, which, to date, have been found to infect only eukaryotes [5].

The nuclear import of viral genomes and the nucleocytoplasmic transport of viral proteins have recently been reviewed in, for example, [6,7,8]. This review presents a brief overview of the literature on host nuclear mRNA export pathways before summarising the diverse mechanisms by which viruses exploit these pathways to complete their replication cycles. We particularly focus on the advances made in studies on retroviruses, DNA viruses, and influenza viruses over the last decades and which have not recently been exhaustively reviewed. Further, this review presents how the research on some of these viruses has contributed to our understanding of the cellular pathways of the nuclear export of mRNAs.

### Nucleocytoplasmic Transport of Macromolecules

The precise evolutionary mechanisms behind the transition from prokaryotes to eukaryotes are still debated. However, it is widely acknowledged that this transition was a gradual change requiring the coevolution of numerous cellular components and mechanisms. One of the more obvious novelties is the NPC, the only gateway connecting the nucleus to the cytoplasm, which allows the selective transition of macromolecules between the two cell compartments [9,10].

**Figure 1:**
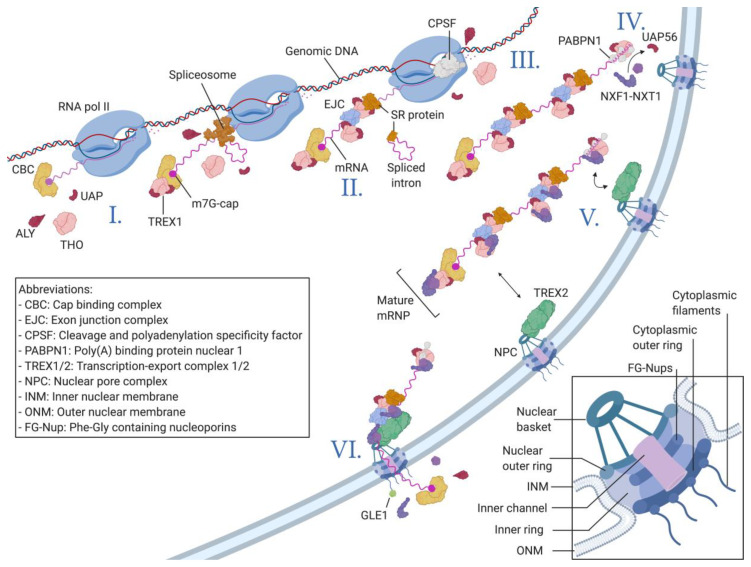
The nuclear RNA processing steps linked to canonical nuclear export. **I.** RNA polymerase II initiates the transcription of genomic DNA (red and blue helix) and produces a capped transcript (purple). The cap is recognized by the cap-binding complex (CBC). The TREX core complex THO and the adapters ALY (Ally of AML-1 and LEF-1) and UAP56 assemble at the CBC. **II.** The spliceosome assembles at the splicing sites of nascent mRNA and promotes the intron excision. The exon junction complex (EJC) is deposited 23 nt upstream of the splicing site where the Serine-Arginine-rich (SR) proteins remain attached to the mRNA. TREX1 interacts with the EJC and SR proteins. **III.** The cleavage and polyadenylation specificity factor complex (CPSF) cleaves the nascent mRNA at the termination sites and promotes the synthesis of the poly(A) tail. **IV.** Nuclear RNA export factor 1 (NXF1)–NTF2-related export protein 1 (NXT1) exportins are recruited by the TREX1 complex starting at the 3′ end of the mature mRNP. **V.** NXF1–NXT1 interacts with TREX2 to direct the mature mRNP towards the nuclear pore complex (NPC) where the mRNP is exported through the interaction of NXF1–NXT1 with FG-Nups inside the NPC pore **VI.** During export, many protein components of the mRNP are removed, while others dissociate from the mRNA in the cytoplasm and are recycled to the nucleus. The square in the bottom right corner shows a more detailed representation of the NPC.

The NPC is formed of multiple copies of 30 distinct proteins called nucleoporins (Nups) and reaches a size of over 110 MDa in humans [11]. Scaffolding Nups form the structural basis of the NPC. At the junction of the inner and outer nuclear membranes sits the inner pore ring forming a continuous channel with a diameter of around 40 nm (Figure 1, zoomed rectangle) [12,13,14]. This inner pore ring connects to a nuclear ring and a cytoplasmic ring from which the nuclear basket and the cytoplasmic filaments respectively emanate [15,16]. Classified separately from the scaffolding Nups are the FG-repeat-containing Nups (FG-Nups), which are named due to their intrinsically disordered domains that are rich in Phenylalanine and Glycine residues [17]. These FG-Nups are anchored to the inner pore ring with the disordered regions protruding into the channel [14,18,19]. The FG-repeats form a dynamic mesh-like structure [20,21] that limits passive diffusion across the pores to molecules of less than 40 kDa or ≈ 5 nm in size [22,23,24]. The transport of macromolecules, such as messenger RNAs (mRNAs), or larger proteins requires the cargo to bind a nuclear transport receptor (NTR). The most common NTRs all possess multiple FG-repeat-binding sites allowing for multivalent but low-affinity interactions with FG-Nups inside the central channel of the NPC. The ultra-fast binding and unbinding dynamics between NTRs and FG-repeats allow the NTR–cargo complex to transition through the NPC in less than 5 ms [25]. How FG-repeats can efficiently plug the pores while still allowing for such fast and specific transport remains a highly intriguing subject that is still widely debated. The most likely model suggests that FG-domains behave like ideal polymers by balancing cohesive and repulsive interactions [26].

The most prominent NTRs belong to the karyopherin family (reviewed in [27]). Within this family importin-α and importin-β are responsible for the nuclear import of proteins (reviewed in [28]), while exportin 1, also known as Chromosome region maintenance 1 (CRM1), allows the export of proteins but also the export of ribosomal subunits and small RNAs [29]. Together, these karyopherins transport over 1000 different cargoes to and from the nucleus [30,31]. The cargo molecules are recognized through specific transport signals, such as the nuclear localization signal (NLS) [32,33], bound either directly by importin-β [34,35] or indirectly by importin-α [36,37], or the nuclear export signal (NES) recognized by CRM1 [38]. The association between karyopherins and their cargoes is modulated by the small GTPase Ran and depends on the phosphorylation state of the bound guanosine molecule. Ran-GTP is concentrated within the nucleus where it stimulates the binding of karyopherin exportins and their NES-containing cargoes [39]. In the cytoplasm, Ran-GDP is predominant and promotes the association between importins and their cargoes [40]. Thus, the gradient of Ran-GTP/GDP across the nuclear envelope is responsible for the directionality of karyopherin-dependent nucleocytoplasmic transport (reviewed in [41]).

The nuclear export of mRNA is somewhat more complicated and tightly connected to the processing of the pre-mRNA. Eukaryotic mRNAs originate as pre-mRNAs that undergo 5′ capping, splicing, and 3′ polyadenylation. During each of these steps, specific proteins remain associated with the mRNA, forming heterogenous messenger ribonucleoprotein complexes (mRNPs) (reviewed in [42]). The export of bulk mRNA is Ran-independent [43], and the implicated NTRs, Nuclear RNA export factor 1 (NXF1, also known as tip-associated protein (TAP)) and its co-factor NTF2-related export protein 1 (NXT1 or p15) can thus not be included within the karyopherin family. Although NXF1 has potential RNA-binding domains, it usually does not bind mRNPs on its own [44]. The recruitment of NXF1–NXT1 to mRNA is, instead, mediated by the transcription–export complex 1 (TREX1) [44]. TREX1 is a dynamic multi-protein complex formed around the THO core complex that binds to mRNPs through adapter proteins such as ALY (Ally of AML-1 and LEF-1; alternatively ALYREF) [45], the U2AF65-associated protein 56 kDa (UAP56) [46], or the UAP56-interacting factor (UIF) [47]. TREX1 is recruited during the processing of pre-mRNAs and thereby directly links the export of mRNPs to each of the co-transcriptional processing steps [48].

The first step in mRNA processing is the synthesis of the 7-methylguanosine (m7G) cap [49] and its association with the cap-binding complex (CBC) [50]. Here, TREX1 is already recruited through the interactions between the Nuclear cap-binding protein subunit 1 (NCBP1), the ALY adapter protein, and components of the THO core complex [51] (Figure 1(I)). This early recruitment of the TREX1 complex is important to orient the nascent mRNP towards the NXF1–NXT1 export pathway instead of the other alternative pathways described later [52]. The presence of TREX1 at this point also inhibits the binding of the CBC by the nuclear exosome targeting complex (NEXT), which can direct the mRNP towards the nuclear degradation pathway [53].

The second major step in mRNA processing is the removal of introns through pre-mRNA splicing. During splicing, the spliceosome, a large ribonucleoprotein complex, assembles on two adjacent exon-intron junctions, which leads to the removal of the intron through the series of mechanisms reviewed in [54]. According to the current consensus, pre-mRNA splicing is the most important determinant for efficient mRNA nuclear export. Different components of the spliceosome, mainly Serine-Arginine-rich (SR) proteins, have been shown to remain bound to the mRNA even after splicing, where they interact with TREX1 adapter proteins [55,56] (Figure 1(II)). Furthermore, the exon junction complex (EJC), which is deposited close to splicing sites after exon removal, has also been shown to interact with TREX1 components [48]. The close link between intron splicing and export is highlighted by the fact that UAP56, a major component of the TREX1 complex, was first identified as a splicing factor and plays an important role in the assembly of the spliceosome [57].

The penultimate step in mRNA processing is the cleavage of the nascent mRNA and the addition of a 3′ polyadenine (poly(A)) tail [58] by the cleavage and polyadenylation specificity factor complex (CPSF) [59], as reviewed in [60] (Figure 1(III)). It has been shown that the activity of CPSF might be the final event allowing TREX1 not only to be recruited to the mRNP but also to efficiently recruit the NXF1–NXT1 exportins [61,62]. Once the polyadenine tail is added, the poly(A) polymerase 1 (PAP1) is released from the mRNA inducing a conformational change within the CPSF [63]. Studies on yeast cells suggest that this conformational change is transmitted through the TREX1 complex to ALY, which induces the departure of UAP56 [61,64], allowing ALY to recruit NXF1–NXT1 and further promoting the export of the now-mature mRNP [65] (Figure 1(IV)).

The resulting mature, correctly processed, and assembled mRNPs are export-competent; they are then released from their respective genes and directed to the NPCs. In metazoan, the translocation from the gene to the NPC seems to follow passive diffusion [66], while in yeast, actively transcribed genes are already located near NPCs through the gene gating mechanism [67,68] (reviewed in [69]). Export-competent mRNPs are concentrated at the surfaces of the NPCs by the TREX2 complex, the main gatekeeper of the NPC [70,71]. TREX2 is anchored to the NPC through the enhancer of yellow 2 (ENY2) and germinal-centre-associated nuclear protein (GANP) [72], which interact with NUP153 and the translocator promoter region protein (TPR), the constituents of the NPC nuclear basket [73]. GANP is also the cornerstone of TREX2′s functions, as it interacts directly with the N-terminal domain of NXF1 [74] (Figure 1(V)). This interaction allows the complex to access the core of the NPC where the NXF1–NXT1 dimer interacts with NUP98, an FG-Nup located at the entry of the pore, followed by interactions with different FG-Nups within the central channel [25] (Figure 1(VI)). Once in the cytoplasm, the NXF1–NXT1-mRNP complex undergoes structural remodeling through the activation of the RNA helicase DEAD box protein 19 (DDX19) via its GLFG-lethal (GLE1) cofactor [75]. DDX19 is already recruited to the mRNP during transcription but remains inactive without GLE1 located at the cytoplasmic filaments of the NPC [76]. The activated DDX19 RNA helicase strips the mRNA of many of its protein partners, the rest of which are later removed by the ribosomes during the pioneering round of translation [77]. 

While optimal mRNA nuclear export is unambiguously linked to the major processing steps of 5′ capping, splicing, and 3′ polyadenylation, recent publications suggest that other co- and post-transcriptional modifications, such as adenine methylation, could also be influential [78,79]. It remains, however, unclear if this influence is due to the direct involvement of the chemical modification itself, the proteins left on the RNA after the modification or the role of m6A in splicing decisions. This topic was reviewed and discussed extensively in [80]. Interestingly, recent work has also suggested that different types of RNAs show different dependencies on NXF1 and TREX components for their export, underlining the complex dynamics within RNA export machinery and suggesting different degrees of redundancy [81].

Although most mRNA is exported through this canonical NXF1–NXT1 pathway, we must consider some alternative cellular mRNA export pathways that can be exploited by viruses. One of these pathways is the alternative RNA export (ALREX) pathway, which is mainly used for the export of intronless mRNAs [82,83]. In ALREX, the export of these mRNAs also relies on NXF1–NXT1 export receptors but requires a reduced number of TREX1 adapters, such as ALY or UAP56, which are mainly recruited during the capping process by the CBC, without the direct involvement of the splicing machinery [84].

Finally, while, the bulk of mRNA exports are karyopherin independent, some specific stress-induced messengers undergo export via the CRM1 pathway (reviewed in [85,86]). Initially thought to be only responsible for the nuclear export of proteins, ribosomal subunits and non-coding RNAs, CRM1 can also bind mRNAs through different specific NES-containing adapter proteins [86]. Among the mRNAs exported by the CRM1 pathway are mRNAs coding proto-oncogenes, cytokines, and lymphokines [85], many of which contain sequences rich in adenine and uracil residues (also called ARE elements) within their 3′ untranslated regions (UTRs) [87] (reviewed in [88]). These sequence motifs are recognized by Human-antigen R (HuR), a ubiquitous adapter protein, which, together with its cofactors acidic leucine-rich nuclear phosphoprotein 31 A and B (ANP32A and ANP32B), can recruit CRM1 to a subset of ARE containing mRNAs [87,89]. HuR and both its cofactors possess different NES motifs, while HuR also has an ARE-binding motif [90]. However, not all transcripts that are exported by CRM1 rely on HuR: The ARE containing mRNA encoding human interferon-gamma (IFN- γ) is CRM1-dependent but HuR-independent [91]. 

CRM1 also plays an important role in the Eukaryotic translation initiation factor 4E (eIF4E) dependent export of mRNAs, which encode proteins involved in cell proliferation and survival, as well as the formation of cancer metastasis [92]. In addition to its cytoplasmic localization, significant amounts of the translation initiation factor eIF4E are present in the cell nucleus [93]. Here, eIF4E allows the export mRNAs that possess specific secondary structures in their 3′ UTR, called the eIF4E-sensitivity element (4E-SE). 4E-SE is approximately 50 nt in length and is bound by the Leucine-rich PPR motif-containing (LRPPRC) [94] adapter protein, which interacts with both eIF4E and CRM1 [95]. In this export mechanism, different components of the NXF1–NXT1 pathway, such as UAP56, DDX3, and the heterogenous nuclear RNP A1 (hnRNPA1), also play a role, while the CBC does not seem to be implicated [94].

## 2. Nuclear Export of Viral mRNAs

Gaining access to the nucleus allows viruses to exploit the nuclear cellular transcription and replication machinery for their own multiplication. Even though the RNAs of these *nuclear viruses* are, apart from influenza viruses, mainly synthesized by the cellular RNA polymerase II, they often harbor features that could be considered aberrant in cellular mRNAs. Contrary to most cellular mRNAs, viruses often make use of partially spliced and/or unspliced mRNAs to increase the coding capacity of their genomes or to ensure their genome replication. In addition to intron retention, viral mRNAs often contain long non-translated regions and premature termination codons. These features are usually considered to be contradictory to the efficient nuclear export of mRNA and detrimental to mRNA stability, inducing their degradation via the nuclear RNA degradation pathway (reviewed in [96]). How viruses avoid the targeting of their RNAs by this surveillance mechanism is beyond the scope of this article, but interesting reviews on this subject can be found in the Viruses Special Issue, “Viral Interactions with Host RNA Decay Pathway” [97,98,99]. To still be able to export all their mRNAs, despite their beforementioned differences to cellular mRNAs, *nuclear viruses* have evolved numerous and diverse mechanisms to exploit the existing cellular export pathways. In the following paragraphs, we present a number of examples from different well-known pathogenic viruses to provide a general overview of the mechanisms used while also illustrating how viruses have contributed to the discovery of the major cellular export mechanisms.

### 2.1. The Success of Retroviruses

Retroviruses are among the most prominent *nuclear viruses*. Named after the mechanism of reverse transcription, which allows them to synthesize a DNA copy of their single-stranded RNA genome, retroviruses insert this DNA copy into the genome of the host cell [100,101,102]. This integration allows the virus to undergo latent infection, during which the proviral insert stays transcriptionally silent [103,104]. During an active infection, however, viral genes from the integrated provirus are transcribed by cellular RNA polymerase II. Simple retroviruses only code the three precursor proteins, Gag, Pol, and Env [105]. Gag is processed into matrix, capsid, and nucleocapsid structural proteins; Pol harbors protease, reverse transcriptase, RNAse H, and integrase functions; and Env produces the envelope proteins responsible for viral tropism [106]. While Env is expressed from a partially spliced mRNA, Gag and Gag-Pol are expressed as polyproteins from unspliced genomic RNA (gRNA) spanning the entire length of the proviral DNA [107]. In addition to the three genes, complex retroviruses code auxiliary proteins harboring important regulatory functions essential for the viral replication cycle [108].

With the discovery of Human Immunodeficiency Virus 1 (HIV-1) as the causative agent of Acquired Immunodeficiency Syndrome (AIDS) in 1983 [109,110], research began on this newly discovered virus family. For a critical review on the discovery of retroviruses and HIV-1, see [111]. While the precise cellular mRNA export pathways were still unknown at the time, it was already demonstrated that intron retention inhibits the nuclear export of mRNAs [112,113]. Thus, an important challenge was to understand how HIV-1 could efficiently export its partially spliced and unspliced mRNAs, which are essential for the expression of its envelope, structural, and enzymatic proteins [106]. Relevant research accelerated in the late 1980s, continued throughout the 1990s, and presently remains ongoing. In the following paragraph, we discuss the breakthroughs in HIV-1 research that not only allowed us to determine how the virus exports its mRNAs but also led to the elucidation of the two major cellular mRNA export pathways.

#### 2.1.1. HIV-1 Research Paved the Way for Nuclear Export Pathway Discovery

HIV-1 is a complex retrovirus that codes six auxiliary proteins expressed from more than 20 different RNA isoforms [114]. The first exported messengers code the auxiliary proteins Tat (Transactivating regulatory protein), Rev (Regulator of expression of viral proteins), and Nef (Negative factor) and are very similar to cellular mRNAs since they are entirely spliced, capped, and polyadenylated. They are thus competent for canonical mRNA export [115]. 

In 1989, Hadzopoulou-Cladaras et al. first observed that the presence of Rev, one of the newly synthesized auxiliary proteins, is necessary for the efficient production of intron-containing mRNAs and the accumulation of structural viral proteins. For its function, Rev requires a *cis*-acting RNA element named the Rev responsive element (RRE), which is localized within the Env coding region [116]. Later that year, the same team determined that Rev increases the stability of the unspliced viral RNAs and contributes to their efficient nuclear export, without any effect on entirely spliced RNAs [117]. The RRE is a 234 nt long and highly structured region, which contains four stem-loops and one branched stem-loop [118]. The in vitro binding of Rev to the RRE was also first described in 1989 [119], and the cooperative binding of multiple Rev proteins to the same RRE was suggested in 1991 [120]. The uncovering of the HIV-1 Rev nuclear export signal (NES) [121] was followed in 1995 by the discovery of the first potential export factor, an FG-repeat-containing protein aptly named human Rev interacting protein (hRIP), which interacts with Rev [122]. However, Rev was also found to interact with numerous other FG-repeat-containing proteins in the yeast two-hybrid system [123]. This and the fact that yeast homolog Rip1p was determined to be a *bona fide* NPC Nup led to the hypothesis that another—at the time undiscovered—protein links Rev to the FG-repeat-containing Nups, with hRIP acting as a carrier to deliver the Rev-RNA-containing complex to the NPC [124]. In 1997, Wolff et al. showed that Rev-dependent nuclear export could be inhibited by Leptomycin B (LMB) treatment [125]. A previously described target of LMB was the yeast Chromosome Region Maintenance Protein 1 (Crm1p) [126]. With this information, Neville et al. demonstrated that Crm1p bridges the interactions between Rev and the FG-Nups of the NPC [127]. Independently, Stade et al. proved that mammalian CRM1, also known as Exportin 1, is an important nuclear export factor [29]. This not only demonstrated that HIV-1 partially spliced and unspliced mRNAs are exported through RRE–Rev–CRM1 interactions but also that CRM1 is the major exportin of NES-containing proteins. CRM1 was later also described as being the major export factor for ribosomal subunits [128]. Interestingly, it was later established that hRIP still plays an important role in HIV-1 RNA nuclear export and is necessary for the correct release of exported HIV-1 mRNA from the exit of the NPC [129].

**Figure 2:**
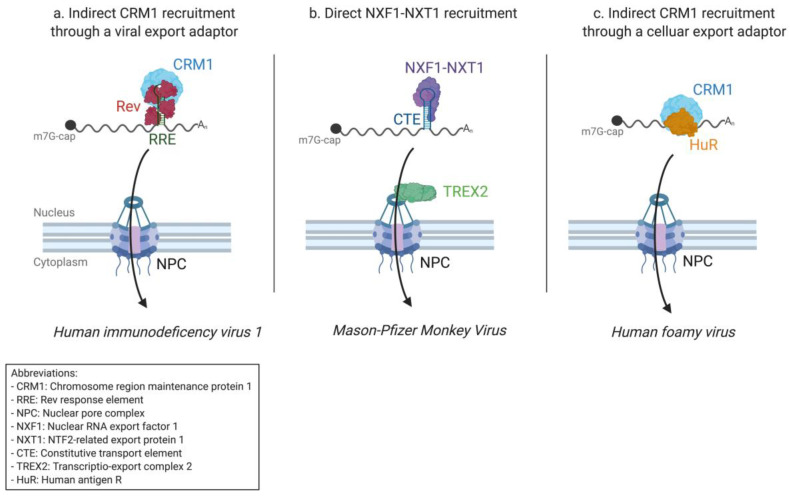
Examples of the mRNA nuclear export mechanisms of retroviruses. (**a**) The export of HIV-1 intron-containing mRNAs occurs via the indirect recruitment of Chromosome region maintenance 1 (CRM1) to the structured Rev responsive element (RRE) RNA element through the multimerized viral Rev protein. (**b**) Mason–Pfizer Monkey Virus intron-containing RNAs are exported through the direct recruitment of cellular nuclear export receptors NXF1–NXT1 to the constitutive transport elements (CTE) secondary RNA structure. For the canonical cellular mRNA export mechanism, NXF1–NXT1 allows the anchorage of the mRNP to the TREX2 complex at the NPC, facilitating its consequent export. (**c**) Intron-containing mRNAs of the Prototype Foamy Virus are exported through indirect recruitment of cellular CRM1 mediated by the cellular adapter protein HuR, which is usually implicated in the nuclear export of ARE-containing cellular RNA.

Thanks to these early advances, the precise mechanism of the Rev-dependent export of intron-containing HIV-1 RNAs is relatively clear today. As soon as the RRE is transcribed and correctly folded, Rev is recruited and oligomerizes co-transcriptionally with the help of the helicase cofactor DDX1 [130]. The oligomerization of Rev exposes multiple NESs that recruit CRM1—this time together with another helicase co-factor, DDX3 [131,132] (Figure 2a). Once assembled, the complex migrates towards the NPC where it is exported in a Ran-GTP dependent manner [133]. In this way, CRM1 is indirectly recruited to the secondary mRNA structure, in this case, RRE, through the intermediary of a viral adapter protein, Rev.

It was recently suggested that the NES of Rev is not the only domain responsible for its interactions with CRM1 [134]. Additional work also demonstrated that different cellular CRM1-associated proteins, such as RNA binding protein 14 (RBM14) [135] and Phosphofurin acidic cluster sorting protein 1 (PACS1) [136], act as Rev co-factors and function in the Rev-mediated nuclear export of viral RNAs. Other cellular proteins, like eIF5a [137] and Src-associated in mitosis protein 68 kDa (Sam68) [138], are also considered to be Rev cofactors and play a role in Rev/RRE functions and HIV-1 replication (reviewed in [139]).

Finally, very recently, Hulver et al. discovered that cellular Tat-specific factor 1 (Tat-SF1) can interact independently of the viral auxiliary protein Tat with the 5′ transactivating response region (TAR) structure and also with additional 5′ regions of the viral genome [140]. These interactions are responsible for the selective nuclear export of viral RNAs, thereby promoting the export of unspliced genomic RNAs but not that of single-spliced RNA isoforms [140].

#### 2.1.2. From HIV-1 to the Simple Mason–Pfizer Monkey Virus

While an important effort was made to uncover the mechanism behind the export of intron retaining mRNA using the HIV-1 Rev/RRE system, knowledge on the canonical export pathway of the bulk cellular mRNA was somewhat lacking. While the importance of splicing was established early on, the nuclear transport receptor remained long undiscovered. In 1994, Bray et al. showed that the insertion of a small element from the Mason–Pfizer Monkey virus (MPMV) genome into unspliced HIV-1 RNAs allows their export and translation in a Rev- and RRE-independent manner [141]. The same observations were also made for a similar RNA element of Simian Retrovirus type 1 (SRV1) [142]. Both MPMV and SRV1 were later defined as being serologically distinct strains of the same Mason–Pfizer Monkey Virus species [143]. As a simple retrovirus, MPMV only encodes the essential Gag, Pol, and Env precursor proteins with no Rev-like protein that could enable the export of the unspliced viral mRNAs. A different mechanism for mRNA export, independent of a viral protein, was thus suspected to exist for simple retroviruses. After the discovery of these RNA elements named constitutive transport elements (CTEs), it was rapidly hypothesized that CTEs could tap into a new RNA nuclear export pathway, potentially the one responsible for the export of cellular mRNA. The essential structure for Rev and RRE substitution is between 150 and 175 nt long, depending on the serotype, and forms a long stem-loop containing two internal loops [144,145,146]. Saavedra et al. showed that when microinjected into *Xenopus* oocyte nuclei, RNAs containing the SRV1 CTE can outcompete the export of cellular mRNA, thus suggesting that retroviral CTE-dependent mRNA export uses the same pathway as the bulk of cellular mRNA [147]. In the same year, Pasquinelli et al. described similar findings for MPMV CTE containing RNAs [148]. Using MPMV CTE RNA as bait for protein capture on an affinity column, Grüter et al. discovered a strongly interacting protein, which they identified as human NXF1 [149], a homolog of the yeast mRNA export factor 67 (Mex67) protein shown previously to be involved in mRNA export [150]. The importance of NXF1 in cellular mRNA export was further uncovered in yeast, where Mex67 was reported to form an export complex with mRNA transport regulator 2 (Mtr2) [151]. In 2000, competition experiments confirmed the role of the homologous NXF1–NXT1 transporter dimer in human cells [152].

Thus, simple retrovirus MPMV, like HIV-1, uses a structural RNA element to export its intron-containing mRNAs. Because it is a simple retrovirus, however, MPMV does not code a viral export protein. Instead, the constitutive transport element directly recruits the cellular export receptor NXF1–NXT1, which allows binding to TREX2 and access to the nuclear pore complex (Figure 2b).

#### 2.1.3. Diversity within the Retroviridae Family

Numerous complex retroviruses use mechanisms very similar to those of HIV-1. Mouse Mammary Tumor Virus (MMTV), Jaagsiekte Sheep Retrovirus 1 (JSRV), and Human Endogenous retrovirus K (HERV-K) all code Rev like auxiliary proteins that bind *cis*-acting secondary RNA structures to direct intron-containing RNA towards the CRM1 export pathway [153,154,155,156]. Interestingly, human T-cell leukemia virus type 1 (HTLV-1) also uses a similar strategy by coding the Rex protein [157,158]. However, the Rex responsive element (RxRE) is located on the 3′ LTR of all viral transcripts [159]. It has been suggested that RxRE plays a role in HTLV-1 mRNA polyadenylation, where the poly(A) signal and actual poly(A) site are unusually far apart [160,161]. Rex has also been shown to interact with viral p30 protein to balance viral gene expression and infection latency [162] (reviewed in [163]).

Rous Sarcoma Virus (RSV) lies between simple and complex retroviruses as it includes a single auxiliary gene *src* of cellular origin providing its oncogenic properties [164,165]. RSV is often considered to be a simple retrovirus since *src* does not have a direct regulatory effect on viral gene expression. Considering the nuclear export of unspliced viral mRNAs, RSV uses a similar strategy to other simple retroviruses. The direct repeat (DR) sequences flanking the *src* gene bind NXF1 and DDX5 [166,167]. Interestingly, however, the RSV Gag protein displays partial nuclear localization and also contributes to the export of intron-containing viral mRNAs in a CRM1-dependant manner [168]. A recent study also succeeded in visualizing the active nuclear export of the viral RNA–Gag complex through the NPC, further highlighting the active role of Gag in unspliced viral mRNA export [169]. Other studies suggest, however, that Gag does not promote the export of DR-containing reporter constructs [167]. Despite this discrepancy, it is possible that RSV exports intron-containing mRNAs via two distinct pathways to direct them towards either Env protein translation or encapsidation. The Gag proteins of several other retroviruses, including HIV-1 and MPMV, also display partial nuclear localization [170,171]. It is, however, still debated how this contributes to the nuclear export of intron-containing mRNAs and their subsequent translation or encapsidation (reviewed in [172]).

Another interesting example is the Murine leukemia virus (MLV), which is often characterized as one of the simplest retroviruses. However, MLV’s strategy for the nuclear export of unspliced mRNAs cannot be considered simple by any means. Contrary to other simple retroviruses, such as MPMV, which use a single *cis*-acting RNA element, MLV has five such elements that are involved in RNA export and are scattered along the entire viral RNA sequence. Some of these elements are found on both spliced and unspliced RNAs (reviewed in [173]). One of these five elements, γ-CTE, which harbors a conserved AAGACA sequence in its stem-loop, directly recruits the NXF1 exportin [174]. The 3′ located cytoplasmic accumulation element (CAE) likely interacts with multiple components of the THO core complex [175]. None of the *cis*-elements identified to date are located within the *env* gene, as is the case with other simple retroviruses. The presence of such numerous distinct elements suggests that MLV mRNA nuclear export is highly regulated and tightly linked to other viral or cellular mechanisms [173]. Moreover, it has been shown that this nuclear export is dependent on some particular components of the TREX1 complex: While spliced MLV mRNAs require the UAP56 helicase for export, unspliced genomic mRNAs instead depend on THOC5, THOC7, and the splice factor SRp20 [175,176]. Recent work showed that MLV RNAs are exported through both the CRM1 and NXF1 pathways, with the CRM1 pathway priming exported unspliced RNAs for packaging [177]. This further proves that nuclear export mechanisms can influence the downstream fate of viral RNAs.

Bridging the gap between retroviruses and the related pararetroviruses, which will be discussed in the following paragraph, is the Prototype foamy virus (PFV) of the *Spumavirinae* subfamily. Foamy viruses (FVs) are considered retroviruses because of their genomic organization and integration of proviral DNA into the host genome. However, they present several significant differences to other retroviruses. For example, FVs’ Pol enzymatic functions are not expressed in a Gag–Pol fusion protein but as separate Pol precursor proteins from a spliced mRNA, and an internal promoter within the *env* gene allows the expression of the *tas* auxiliary gene [178,179]. Another feature unique to FVs is that for 5 to 10 % of FV particles, reverse transcription of the packaged genomic viral RNA already starts in the preassembling capsids [180]. This means that the released viral particles contain both genomic RNA and genomic DNA [181]. Furthermore, the budding of preassembled FV capsids takes place not only at the plasma membrane but also in the intracellular compartments (endoplasmic reticulum and Golgi) [182]. When it comes to the export of mRNA, FVs are still faced with the same challenges of the nuclear retention of unspliced mRNAs. Here, FVs seem to combine the two major pathways used by other retroviruses. FV unspliced mRNA export is viral-protein independent but relies on the cellular CRM1 export pathway [183]. Unlike other retroviruses, CRM1 is thus not recruited to viral RNA by a viral protein but rather by the host adapter HuR, which is also implicated in the export of cellular ARE-containing RNAs [90,183] (Figure 2c). It has been suggested that other adapter proteins (ANP32A and ANP32B) from the same pathway are also involved, bridging HuR to CRM1 [183]. Foamy viruses are often referred to as the oldest retroviruses [184], suggesting that the HuR export pathway might have paved the way for the evolution of Rev-like and CTE-dependent strategies. For other retroviruses, the FV Gag protein is partially located within the nucleus, and correct viral egress is dependent on this nuclear localization [185], further suggesting that Gag is involved in the sorting of unspliced mRNA for protein synthesis or virion assembly [186].

### 2.2. mRNA Nuclear Export of Pararetroviruses

Pararetroviruses, like retroviruses, replicate their genomes by reverse transcription but do not integrate their genomic DNA into the host genome. Like for foamy viruses, reverse transcription occurs late in viral replication, leading to the encapsidation of a partially double-stranded DNA, also called relaxed circular DNA (rcDNA) [187]. 

The only pararetrovirus known to infect humans is the Hepatitis B virus (HBV), a member of the *Hepadnaviridae* family of strictly hepatotropic viruses. Only 3.2 kbp in size, HBV has the smallest DNA genome of all known mammalian viruses [188]. Upon infection, rcDNA is transported to the nucleus where the host machinery converts it into covalently closed circular DNA (cccDNA) [189]. This cccDNA then associates with cellular histones to form a mini-chromosome, which persists within the host nucleus [190]. Due to its small size, the HBV genome has a very high coding density with four overlapping genes. To efficiently express each of these genes, HBV relies on four promoters, two enhancers, and a single termination and polyadenylation site leading to the synthesis of six mRNAs (reviewed in [191]). Aside from being used as a template for reverse transcription, pregenomic RNA (pgRNA), which is slightly longer than the genome, is also a messenger for polymerase and core protein expression. The precore mRNA (preC RNA) facilitates the expression of the Hepatitis B e antigen (HBeAg) [192]. The subgenomic mRNAs PreS1 and PreS2, which are 2.3 kb and 2.1 kb in length, respectively, code the three different variants of the surface antigens L, M, and S [193]. The last subgenomic mRNA is rather small (0.7 kb) and codes the X regulatory protein [194]. The long pg and preC mRNAs can be spliced, resulting in mRNAs coding the HBV splice-generated protein (HBSP) [195], which has been linked to HBV pathogenesis [196]. Overall, HBV transcripts can be conceptually divided into three groups: unspliced pg and preC mRNAs, spliced pg and preC mRNAs, and intronless subgenomic mRNAs. Each of these isoforms are exported to the cytoplasm either for translation or for encapsidation and reverse transcription [197].

**Figure 3:**
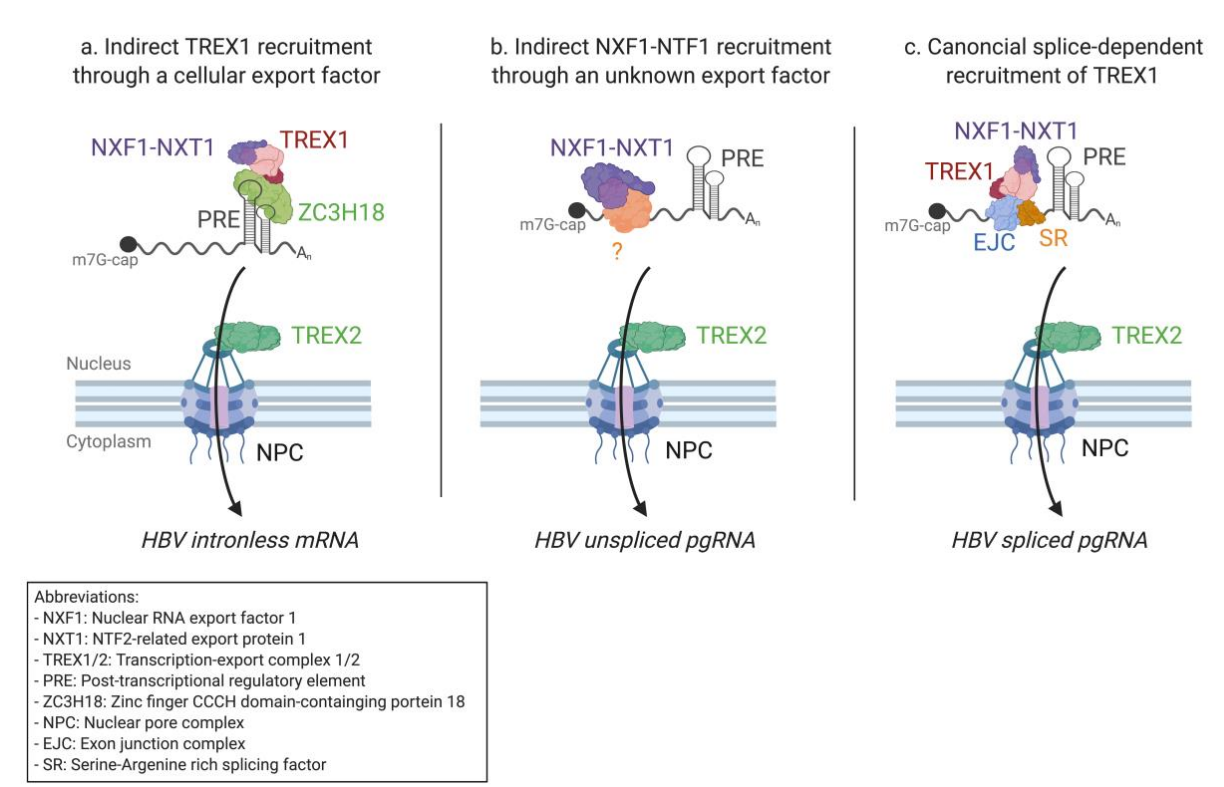
Hepatitis B Virus mRNA nuclear export by the NXF1–NTF1 pathway. (**a**) Hepatitis B virus intronless subgenomic mRNAs are exported through the association of the TREX1 complex to the PRE element via the cellular adapting protein ZC3H18. As is the case for the canonical mRNA export pathway, TREX1 recruits NXF1–NXT1 nuclear transport receptors allowing export through the NPC after interaction with the TREX2 complex. (**b**) Unspliced pgRNA is exported through the recruitment of NXF1–NXT1 in a PRE- and Core-independent manner. (**c**) Spliced Hepatitis B virus (HBV) pgRNAs are exported in a splicing-dependent manner, most likely through the canonical cellular mRNA export pathway.

All HBV transcripts share a 365 nt long *cis*-acting RNA element, the posttranscriptional regulatory element (PRE), which overlaps the X coding sequence [198]. After PRE’s discovery in 1993, it was rapidly proposed that PRE might play an important role in mRNA nuclear export, as is the case for HIV-1 RRE and MPMV CTE [199]. Interestingly, however, the PRE-linked export mechanism is CRM1-independent and also functions differently from MPMV CTE-dependent export, as the PRE is unable to directly bind the NXF1–NXT1 export receptors [200]. The PRE can be divided into two export relevant regions, the sub-elements PRE 1 and 2 (SEP1 and SEP2), each forming multiple stem-loop secondary structures [201]. Several PRE interacting proteins have been identified and proposed to play a role in the nuclear export of HBV mRNAs. These proteins include glyceraldehyde-3-phosphate dehydrogenase (GADPH), Polypyrimidine tract-binding (PTB) protein, and the La protein [202,203,204]. The precise role of each of these proteins in HBV mRNA export remains unclear [205]. A much more promising candidate is the zinc finger CCCH domain-containing protein 18 (ZC3H18), which has been found to bind SEP1 and to recruit several components of the TREX complex, including THOC2 [206] (Figure 3a). Once the TREX1 complex is recruited, intronless subgenomic mRNAs are presumably exported through the NXF1–NXT1 pathway.

Unspliced variants of HBV pgRNA and preC mRNAs are exported in a PRE-independent manner, suggesting that HBV might, like HIV-1, use different pathways to export different mRNA isoforms [199]. It has been shown that the HBV core protein displays nucleo-cytoplasmic shuttling properties [207] and interacts in the nucleus with pgRNAs forming pgRNPs [208]. Core also interacts with TREX components and NXF1–NXT1 export receptors [209]. The nuclear export of pgRNA is, however, independent of the core protein [209] (Figure 3b). Recent research identified a new HBV RNA binding protein involved in RNA nuclear export, the TAR DNA-binding protein (TARDBP). TARDBP has been suggested to play a passive role in mRNA nuclear export by inhibiting the splicing of pgRNA, thus favoring the export of unspliced pgRNA isoforms [210]. Hu et al. also recently determined that UAP56 interacts with the HBV X protein, potentializing the export of single-exon HBV mRNAs in cell cultures [211]. This supports the hypothesis that the export of spliced HBV mRNAs is linked to splicing even if HBx does not interact directly with NXF1 or NXT1 (Figure 3c). In summary, HBV likely exports all its different mRNA isoforms through an NXF1–NXT1-dependant mechanism. However, only the export of intronless subgenomic mRNAs depends on the PRE *cis*-element. Recent research suggests that pregenomic and precore RNA export is partially regulated by splicing factors. Although the export of HBV transcripts has been studied for nearly 30 years, important details on its precise mechanisms remain elusive. Understanding the export mechanisms used by HBV could lead to the development of new and specific antiviral components needed in the pursuit of Hepatitis B control and prevention (reviewed in [212]).

### 2.3. Nuclear Replicating DNA Viruses

Apart from the previously discussed pararetroviruses and some other exceptions, such as *Poxiridae* and *Megaviridae*, most DNA viruses replicate their genomes within the nucleus of the host cell. Most such viruses use complex mechanisms to regulate their gene expression from an early phase, which provides the proteins needed for genome replication, to the late expression of the structural proteins needed to package DNA and form virions (reviewed in [213]). Although most nuclear DNA viruses undergo splicing for most of their transcripts, some mRNAs retain at least one intron that hampers their export via the canonical cellular mRNA export pathway. Thus, even though they do not require the export of a genomic or pregenomic RNA like retroviruses and pararetroviruses, DNA viruses have also had to evolve mechanisms to guarantee the efficient export of their intron-containing mRNAs. After providing an overview of the mechanisms used by two large DNA virus families, *Adenoviridae* and *Herpesviridae*, for which experimental data on this topic are plentiful, we will also discuss the historical and current findings concerning human oncogenic *Papilloma* and *Polyomaviridae*.

#### 2.3.1. Adenovirus mRNA Nuclear Export

Adenoviruses (AdVs) are non-enveloped viruses with a linear double-stranded DNA genome of about 36 kbp. They were first discovered in adenoid cell culture, hence the name (*adénos*, gland) [214]. Adenoviruses are ubiquitous, and infection is usually asymptomatic or mild and self-limiting in immunocompetent hosts. However, in immunocompromised individuals, adenoviral infection can lead to more severe illness [215].

The human Adenovirus genome is rather dense, with over 40 genes [216]. To assure efficient viral replication, these genes are expressed in an orderly manner to reprogram the host cell towards virus replication without alerting the immune system or inducing cell damage, leading to apoptosis [217]. The first gene expressed is the early gene 1A (E1A) [218], which drives the expression of other early proteins originating from the E2, E3, and E4 transcription units [219]. Later during infection, the expression profile switches gradually towards the major late promoter (MLP), inducing the synthesis of the late gene products necessary for the assembly of the viral progeny [219]. Overall the transcriptional program of AdV is highly regulated by different mechanisms, one of which is alternative splicing, leaving individual transcripts either partially or entirely unspliced [220]. Some viral transcripts are also specifically retained in the nucleus until the appropriate time for their expression [219].

The study of AdV mRNA export mechanisms has, to date, mainly focused on late messengers, and the situation seems rather complex. A potential role of the viral E1B-55k protein in the export of late AdV transcripts was first observed in 1986 [221]. However, its role in export was suggested to be rather indirect and upstream of the actual export event [222]. E1B-55k is, however, the chief orchestrator of the events leading to late AdV mRNA export by first reorganizing, together with the viral protein E4orf3, promyelocytic leukemia (PML) nuclear bodies at the periphery of which viral transcription and replication occur [223,224]. At later stages of infection, another viral protein, E4orf6, enters into competition with E4orf3 and induces the relocalization of E1B-55k towards the periphery of the nuclear speckles, a storage place for splicing factors and mature, export-competent, viral, and cellular transcripts [224]. All these sequential virus-induced modifications of nuclear structures inhibit the maturation and export of cellular mRNAs while promoting the export of intron-containing viral transcripts [225]. Furthermore, E1B-55k, E4orf6, and some cellular components forming a ubiquitin-3-ligase complex are required for the efficient transport of late mRNAs towards the cytoplasm [226]. The relevant mechanism relies on the inhibition of the cellular components essential for the degradation of intron-containing mRNAs [227]. For the many other roles of E1B-55k in the regulation of Adenovirus gene expression, see [228].

**Figure 4:**
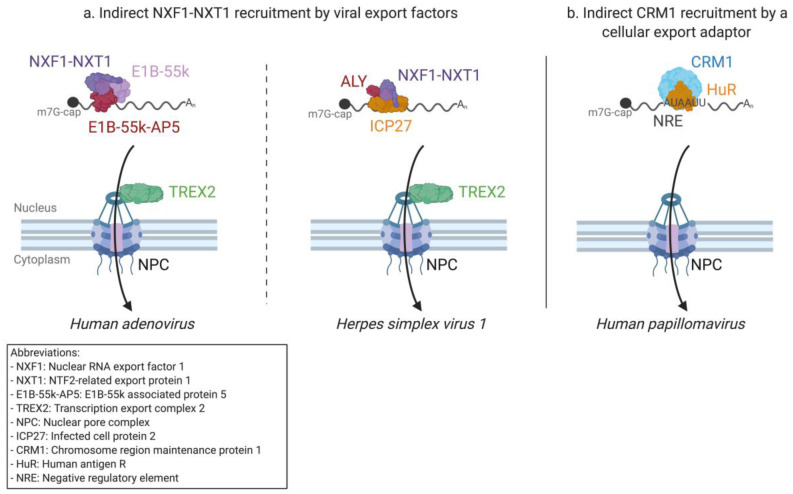
Examples of the mRNA nuclear export of DNA viruses. (**a**) Both Adenoviruses and Herpesviruses recruit NXF1–NXT1 exportins to their mRNAs indirectly through viral adapter proteins. The Adenovirus early protein E1B-55k and its associated cellular partner, E1B-55k-AP5, bind to viral mRNAs and recruit NXF1–NXT1 which promotes nuclear export of the mRNP complex through the TREX2 NPC interaction mechanism. Herpes simplex virus 1 codes the ICP27 export protein, which is well conserved within *Herpesviridae*. ICP27 recruits NXF1–NXT1 export receptors through the cellular ALY protein, inducing transport through the NPC by interacting with the TREX2 complex. (**b**) Human Papillomavirus late mRNAs presumably recruit cellular CRM1 through the cellular adapter protein HuR, which binds to the negative regulatory element (NRE) RNA element.

The current model of export for AdV late mRNAs suggests the participation of the cellular E1B-55k-associated protein (E1B-55k-AP5), which interacts with both the viral mRNAs and NXF1 [152,229,230] (Figure 4a). In this model, E1B-55k could play a role in the release of late mRNAs from their sites of transcription, but the precise molecular mechanisms by which E1B-55k and E1B-55k-AP5 lead to the selective export of AdV mRNAs remain unclear.

While the roles of Adenoviruses in human oncogenesis are still debated, there have been numerous attempts at using viruses to target cancer cells [231,232,233] (reviewed in [234]). The most challenging part of this approach is to modify a virus in a way that it becomes specific to cancer cells while not affecting normal cells. In many types of cancer, the export mechanisms of mRNAs are highly deregulated, and the retention of intron-containing mRNAs is no longer efficient [235]. Thus, using modified viruses, which are unable to export their mRNAs from the nucleus under normal conditions, is a promising approach. Indeed, E1B-55k-deficient Adenoviruses have a selective oncolytic effect in combination with cisplatin, when compared to the use of the classic anti-cancer drug alone [236]. It was later demonstrated that the selectivity of the virus could be attributed to inefficient viral mRNA export in healthy cells [235].

#### 2.3.2. Herpesvirus mRNA Export—A Shared Strategy with Different Tools

*Herpesviridae* are a large family of viruses that induce numerous pathologies in a wide range of hosts. They have a sizeable linear dsDNA genome enclosed in an icosahedral capsid surrounded by a proteinaceous tegument layer and a lipidic membrane [237]. Herpesviruses are unusual as they can undergo latent infection and reactivate at a later stage [238]. The nine different herpesviruses infecting humans are classified into three different subfamilies, *Alphaherpesvidinae*, *Betaherpesvirinae*, and *Gammaherpesvirinae* [239]. Herpesviruses are ubiquitous and present in around 80% of the human population [240]. Despite their large genomes, they possess very few intronic sequences, with up to 98 % of their genes being intronless. Although new splice sites have recently been mapped to the genome of Herpes simplex virus 1 (HSV1 or Human alphaherpesvirus 1), their functions remain to be confirmed [241]. Herpesviruses thus need splicing independent mRNA export mechanisms. A viral mRNA export factor has been discovered within each of the three subfamilies, and most experimental data on the precise mechanism by which they function are available for HSV1 and its infected cell protein 27 (ICP27).

The role of ICP27 as an export adapter for viral mRNAs was first suggested after the discovery of its nuclear localization, RNA binding, and nucleo-cytoplasmic shuttling [242,243,244,245,246,247]. ICP27, which itself is expressed from a spliced mRNA, gives HSV1 intronless transcripts access to the canonical mRNA export pathway by interacting with ALY, NFX1, and cargo mRNAs [248,249,250] (Figure 4a). Although it has been suggested that ALY is dispensable for HSV1 export [250], its presence significantly improves efficiency, likely by adding specificity to ICP27s’ interactions with the target mRNAs [251,252,253]. While the RNA binding region of ICP27 was mapped to its RGG motif early on [243], no RNA sequence specificity for this interaction was found, and the RNA elements necessary for ICP27-dependent export remain thus undiscovered. Several other proteins have been found to interact with ICP27, including the splicing factor UIF and nucleoporin Nup62 [254,255]. While the latter interaction could compete with cellular export, their precise roles in RNA export are not currently known. Finally, ICP27 has also been suggested to be involved in splicing and polyadenylation and could be linked to the switch from a latent to a lytic infection [241].

The viral export factors within the three herpesvirus subfamilies share an overall amino acid homology of 20 to 30 % and a C-terminal homology of 40 to 60 % with ICP27 [256,257]. They also all share many other common features, such as nucleocytoplasmic shuttling, RNA binding activity, and the requirement for phosphorylation by cellular kinases. However, the kinases responsible for this phosphorylation vary from one subfamily of *Herpesviridae* to another, as does the nature and position of the RNA binding domain (reviewed in [258]). These small but significant differences are exemplified by the fact that neither HSV1 ICP27 nor the Human cytomegalovirus’s unique long 69 gene product (UL69) can entirely complement the mutant Epstein–Barr virus EB2 protein, which is essential for viral mRNA export and virus production [259]. Overall, all the herpesvirus export protein homologs share a common mechanism allowing the export of viral mRNAs by forming ribonucleoprotein complexes with components of the TREX complex and the cellular export factor NXF1.

#### 2.3.3. Small DNA Virus mRNA Export

Papilloma- and Polyomaviruses are both small dsDNA viruses that historically belonged to the same *Papoviviridae* family. Although they were split into two distinct families, the International Committee on the Taxonomy of Viruses (ICTV) recently included these two families within the same *Papovaviricetes* class due to their numerous similarities and phylogenetic proximity [260]. Both families include viruses that have oncogenic potential, some of which are associated with human cancers, particularly with cervical, skin, prostate, and colorectal cancer [261]. Viral gene expression is finely regulated during an early and a late expression phase, and, in both cases, alternative splicing is an essential regulatory mechanism [262,263,264,265] (reviewed in [266] and [267]). These alternative splicing events lead to the production of intron-retention or entirely unspliced transcripts that need to overcome nuclear retention to be exported to the cytoplasm for translation.

The most common human polyomaviruses are the BK polyomavirus (BKPyV) and JC polyomavirus (JCPyV), bearing the names of the patients from whom they were first isolated [268,269]. Both these viruses are ubiquitous among the human population without any clinical significance in healthy individuals. However, in immunocompromised settings, both can lead to severe disease. Further, their clinical significance has risen in recent years, starting with the global AIDS epidemic and continuing today with a growing number of immunocompromised organ receivers and gene therapy patients [270,271,272,273]. The model species of polyomaviruses is simian virus 40 (SV40). Interestingly, SV40 also circulated in the human population after contaminated oral polio vaccines were administrated to millions of people between 1954 and 1961 [274]. Polyomaviruses are highly cancerogenic in non-permissive hosts. However, the implications of SV40 in human cancers are still debated to date [275,276,277,278,279] (reviewed in [280]). Little is known about the precise mRNA export mechanisms used by human polyomaviruses. It was, however, due to experiments with SV40 that the close relationships between splicing and mRNA export were first suggested, which are now widely acknowledged [281]. Similar information on polyomavirus mRNA export came from studies on the murine polyomavirus (MuPyV) performed in the 1990s, showing that the 5′ splice site, which is retained in the unspliced late viral protein 2 (VP2) mRNA, influences its nuclear export [282]. Interestingly, it was also demonstrated that while the strength of the splice site is inversely proportional to export efficiency, splicing itself does not increase the amount of exported mRNAs [283]. It is probable that splicing factors bind this suboptimal splice site, and, even though they do not lead to splicing, they might interact with some components of the export machinery and thus slightly promote the inefficient and leaky export of this unspliced mRNA.

The knowledge about the role of Papillomaviruses in human tumors is well established. HPV is most commonly known as the causative agent of cervical cancer in women but can also cause, among others, penile and anal cancer, as well as head and neck cancer, alongside more benign genital and cutaneous warts. According to the centers for disease control and prevention (CDC), HPV infection is the most common sexually transmitted infection today [284]. Early and late HPV gene expression is not only regulated over time but is also highly dependent on the differentiation process of the infected epithelial cells [285,286,287,288]. Initial infection occurs at the basal layer of the epithelium [289], where the early phase of gene expression is centered on viral genome maintenance, the stimulation of cell division, and apoptosis inhibition, which all allow viral genomes to spread among the dividing cells [290]. Infected daughter cells migrate upwards through the various epithelial layers while completing keratinocyte differentiation [291]. Cellular differentiation leads to tightly regulated changes in viral gene expression, each of them linked to a particular epithelial layer [292,293,294]. Late viral genes are only expressed in the upper layers of the epithelium, where they lead to the production of structural proteins and the assembly and release of new viral particles [295,296,297]. The regulation of gene expression is orchestrated by polyadenylation and alternative splicing [298] (reviewed in [299]). Recent advances also suggest an essential role of cellular DNA damage response in the regulation of late HPV gene expression [300]. Interestingly, even if late transcripts are first detected in mid-layer epithelial cells, fully processed and exported mRNAs are only present in the uppermost layers [301,302,303,304]. This observation suggests that, although they are transcribed earlier on, the processing and export of late mRNAs also depends on the differentiation of keratinocytes.

The regulation of late gene expression is further linked to an AU-rich late negative regulatory element (NRE), present on all but one late transcript [305]. As the name implies, the NRE has an inhibitory effect on late HPV gene expression [306]. Reporter assays have shown that the inhibitory effect of this regulatory element can be overcome via the addition of HIV-1 RRE to the reporter transcript while providing the HIV-1 Rev protein *in trans* [307]. This suggests the role of mRNA nuclear export or retention in the regulation of late gene expression. Several cellular proteins were found to bind the NER [308], and one of these proteins was later identified as HuR [309], which is essential for the export of ARE-containing mRNAs [90] (Figure 4b). Further underlining the potential role of HuR is the fact that upon the viral-induced proliferation of the epithelial cells, HuR is relocated from the nucleus to the cytoplasm [310]. The expression of late genes L1 and L2 is also correlated to the overexpression of HuR in cell cultures [311]. If the HPV late gene expression is indeed linked to viral mRNA export by HuR through the ARE-RNA export pathway, one might expect LMB treatment to abolish this effect by inhibiting the CRM1 exportin. HPV infected cells have been treated with LMB, but this treatment rapidly induced apoptosis linked to the expression of viral protein E7 [312]. The effect of this treatment on HPV late gene expression and mRNA export was, therefore, not tested. Thus, the role of HuR and the ARE containing RNA export pathway in the nuclear export of late HPV transcripts still needs to be clarified.

### 2.4. The One Exception—The Nuclear Export of Influenza Virus RNAs

We have seen that all reverse transcribing—and most DNA—viruses access the cell nucleus to exploit cellular transcription and replication machinery. Conventional RNA viruses code their own RNA-dependent RNA polymerase (RdRp) and are thus able to replicate their genomes within the cytoplasm without requiring direct access to the nucleus. However, while many RNA viruses only interact with the nucleus through the import of viral proteins that affect host gene expression, there is one known exception to this rule: influenza viruses. Influenza viruses have a segmented negative single-stranded RNA genome (with eight segments for influenza A viruses (IAV)), which is replicated with high mutation rates and has the potential for the re-assortment of the segmented genomes between different viral strains [313,314]. This genomic variability leads to high antigenic variability requiring annual vaccinations. Sporadically, the emergence of a new IAV strain due to an antigenic shift can lead to pandemics and high mortality due to a lack of immunity conferred by vaccination or prior infections [314,315,316]; for reviews, see [317,318].

Influenza virus RdRps lack methyltransferase activities and are unable to cap the 5′ ends of viral mRNAs, which is required for their proper processing and function. Thus, during a process called cap-snatching, the influenza virus RdRp binds to the C-terminal domain of cellular RNA polymerase II and cleaves the newly synthesized cellular mRNA 10–13 nucleotides downstream of the 5′ cap [319,320,321] (reviewed in [322]).

Among the eight genomic segments of IAV, the viral RdRp transcribes segments 1 to 6 into six intronless mRNAs, while segments 7 and 8 are transcribed into mRNAs that can undergo alternative splicing [323,324,325]. Viral RdRp is also responsible for the replication of the viral genomic segments that also takes place within the host cell nucleus [326,327]. Thus, four different types of viral RNAs are synthesized during the IAV replication cycle: intronless mRNAs, spliced isoforms of intron-containing mRNAs, unspliced intron-containing mRNAs, and newly produced viral genomic segments, which are associated with the viral polymerase complex and are encapsidated by the viral nucleoprotein (NP) forming the eight viral ribonucleoproteins (vRNPs) [328,329,330].

#### 2.4.1. Export of Influenza A Virus mRNAs 

Empirically, one might expect all intronless viral mRNAs to be exported through the ALREX pathway. However, the export of the different intronless viral mRNAs is differentially affected by the depletion of NXF1, the major export receptor involved in the ALREX export pathway [331]. Surprisingly, the export of early transcripts coding the three polymerase subunits PA, PB1, and PB2 is entirely insensitive to NXF1 depletion but also to LMB inhibition of the CRM1 pathway [331]. These findings suggest that early influenza transcripts use a unique nuclear export mechanism independent of NXF1 and CRM1. Unlike intronless polymerase subunit transcripts, intronless mRNAs transcribed later during the infection cycle of IAV display, at least for some of them, sensitivity towards NXF1 depletion [331]. Under this condition, the export of mRNAs coding neuraminidase (NA) and haemagglutinin (HA) is compromised, whereas the NP coding mRNA remains unaffected [331]. Larsen et al. showed that the effect of NXF1 depletion on influenza mRNA export was different from one cell type to another and that NP mRNA export was, indeed, NXF1-dependent in human epithelial lung cells (A549) [332]. Since these cells reflect in vivo IAV infection more accurately, it is reasonable to consider NP mRNA export as comparable to the NXF1-dependent export of HA and NA mRNAs.

It is important to highlight that despite the fact that all IAV mRNAs undergo capping and polyadenylation, two processes that are involved in the recruitment of the NXF1–NXT1 nuclear export machinery, these export receptors are used only by some of the IAV mRNAs. This observation can be explained by the fact that the mechanisms by which IAV mRNAs are processed significantly differs from the canonical cellular mechanisms. Although NCBP1 has been reported to be involved in IAV mRNA export, it is still unclear whether the CBC might be disturbed during cap-snatching, thus interfering with its canonical role in RNA export [51,333]. Furthermore, the poly(A) tail is not synthesized by the PAP but rather by viral RdRp stuttering on the poly(U) stretch before its dissociation from the template RNA [334]. This implies that CPSF and PABP cannot play their usual roles in the recruitment and activation of the TREX1 complex. It has, however, been shown that the efficient export of viral mRNA requires ongoing Polymerase II activity [335], suggesting that viral transcription likely takes place in an environment allowing for at least some of the interactions that usually link mRNA export to processing to occur. Further complicating our current understanding of IAV intronless mRNA export is the fact that a poly(A) tail is required for the efficient export of cellular intronless mRNA by the ALREX pathway [83]. Whether or not the atypical polyadenylation of IAV mRNAs can contribute equally towards this mechanism remains unknown.

Since the mRNAs transcribed from segments 7 and 8 can be spliced to produce the non-structural proteins NS2 and NS3 and matrix proteins M2, M3, and M4, respectively [323,324,325], one might expect these spliced mRNAs to be exported by the splicing-dependent NXF1–NXT1 export receptors. This is indeed the case, but, surprisingly, the unspliced mRNAs coding the NS1 and M1 proteins seem to follow a similar pathway [331]. Recent studies have shown that the precise mechanism might deviate from the canonical pathway, at least for the export of M coding mRNAs as the splicing of the M1 mRNA has been shown to occur post-transcriptionally at the nuclear speckles [336]. Viral NS1 protein and its cellular partner, the NS1 binding protein (NS1-BP) [336], are also involved in the splicing of M1 mRNA, as well as in the export of both the spliced and unspliced forms of M mRNAs [337,338]. This suggests that the nuclear accumulation of NS1 is responsible for the M1 transcripts’ relocalization towards the nuclear speckles where they undergo splicing before being exported towards the cytoplasm, leading to the subsequent accumulation of the M2 protein later during infection. This temporal regulation is essential since high levels of M2 protein are cytotoxic and only required during the later phases of the IAV replication cycle [339]. Another critical component identified for M mRNA nuclear export is UAP56 [331], which co-localizes with NS1, further corroborating its importance in the mechanism [340].

Interestingly, recent work showed that the knockdown of cellular NS1-BP leads to a significant reduction in M mRNA export without altering cellular bulk mRNA export [336]. This suggests that IAV M mRNA speckle-dependent nuclear export is unique and thus a highly attractive target for the development of new antiviral agents [341]. Surprisingly, like for the export of intronless mRNAs, the mechanism of M mRNA nuclear export is not the same as that of the NS mRNAs. It has been previously shown that NS2 mRNA export is insensitive to UAP56 depletion [331] and that there are no temporal differences in the accumulation of NS1 and NS2 proteins. In addition, the splicing and export of NS mRNA are independent of the NS1 protein [342]. Further emphasizing the differences in the nuclear export of M and NS mRNAs is the fact that a chemical component that inhibits M mRNA export only has mild effects on NS mRNA export [341]. Contradicting the presumably distinct splicing and export mechanisms of the M and NS mRNAs is the involvement of the RNA helicase eIF4A3 in the splicing and export of both RNAs [343]. Other cellular RNA helicases are also involved in IAV mRNA export. For instance, it has been shown that DDX19 is associated with viral polymerase and is involved in the export of all viral mRNAs. Moreover, its involvement can be bypassed during the later phases of infection [344]. This suggests that IAVs have evolved some redundancies in their viral mRNA nuclear export, which might explain the difficulties in proposing a single model for the mechanisms involved.

#### 2.4.2. Export of Influenza A Virus vRNPs

As mentioned before, influenza viruses export their replicated genomic segments into the cytoplasm as vRNPs, in which negative viral RNAs are encapsidated by the NP protein and associated with the polymerase complex (reviewed in [345]). The export of these vRNPs depends on the NS2 viral protein [346], also known as the Nuclear Export Protein (NEP), and M1 [347,348]. Both NEP and M1 are associated with purified virions [349,350]. While a daisy chain model of interactions was first proposed where M1 binds NP and NEP binds M1, the current model suggests that M1 and NEP interact with each other and that both also interact with NP. NEP possesses a canonical and an atypical leucine rich NES, which are both involved in the CRM1-dependent export of vRNPs [351,352,353]. Yeast and mammalian two-hybrid experiments recently showed that NEP interacts with Nup98 and Nup214, two nucleoporins that are essential for IAV nuclear export and replication [354,355]. Interestingly, NP itself also possesses several NES motifs and displays CRM1-dependent nucleo-cytoplasmic shuttling [356,357]. NP also interacts with NXT1, co-factor of the major mRNA export receptor, NXF1 [358]. Surprisingly, interaction with NXT1 stimulates the CRM1-dependent nuclear export of NP (and probably also that of vRNPs) [358]. While these findings suggest a novel redundancy in the IAV replication cycle, here centered on the nuclear export of vRNPs and the different viral proteins that are able to recruit the export machinery, the precise role of NP in nuclear export remains unclear. A recent study also suggested the influence of cell polarity on influenza vRNP nuclear export [359].

The nuclear export of both IAV mRNAs and vRNPs has been explored as a potential target for anti-influenza treatment strategies [341]. Overall, the precise nuclear export mechanisms of all types of IAV RNAs remain unclear due to many contradictory results, which might be due to the use of different cell types or differences between the viral strains. Recent research suggests that the impaired nuclear export of vRNPs might be responsible for host restrictions between avian and human influenza [360], further underlining the need for a better understanding of these mechanisms.

## 3. Concluding Comments

Viruses that require access to the nucleus of their eukaryotic host cells are faced with numerous challenges. One of these is the requirement for the nuclear export of their different mRNA isoforms, which are essential for the viral replication cycle. The cellular mechanism for mRNA export is highly dependent on proper pre-mRNA processing, including 5′ capping, 3′ polyadenylation, and, most importantly, intron splicing. However, many viral transcripts retain introns and contain other features often considered contradictory to efficient RNA export, such as long non-translated regions harboring premature stop codons.

To overcome these obstacles, viruses have evolved different strategies to exploit the cellular pathways of mRNA nuclear export. The precise mechanisms of export are highly variable between different viral families, and, in some instances, even within the same family. A common characteristic among them, however, is their dependency on the formation of ribonucleoprotein complexes, including nuclear export receptors. These complexes can be formed in many different ways. In some cases, like MPMV, nuclear transport receptors are recruited directly to viral mRNAs through a secondary RNA structure. In other cases, an adapter protein of cellular or viral origin, provides the link between the viral mRNA and the nuclear export receptor. The resulting ribonucleoprotein complexes can be relatively simple, but, more often, they are complex and very dynamic. 

The CRM1 export pathway is exploited to export partially spliced and unspliced mRNAs, particularly in complex retroviruses that code for an adapter protein recruiting CRM1 on the secondary structures of viral mRNAs. The nucleolar localization of these adapter proteins is probably related to that of CRM1 but also to the escape of viral intron-containing mRNAs from the host surveillance machinery. It is important to highlight that CRM1 is also the major exporter of proteins (cellular and viral) and is systematically over-expressed and deregulated in cancer cells, leading, for example, to the abnormal accumulation of tumor suppressor proteins in the cytoplasm and their functional inactivation (for a review, see [361]). For this reason, research on, and the identification of, effective and reversible CRM1 inhibitors, called SINE (selective inhibitor of nuclear export), has been very active for many years, and several molecules have already been tested and selected after phase I, II, and III clinical trials. One of them, selexinor, was approved in the United States in 2019 by the Food and Drug Administration (FDA) [362]. The specific inhibition of CRM1 is, of course, also deleterious to viral replication and could, therefore, represent an effective antiviral treatment. As we have seen, CRM1 inhibition not only considerably reduces the replication of several influenza viruses (H1N1, H3N2, H5N1, and H7N9) but also that of HIV-1 by blocking the export of its genomic RNA and the production of new viral particles [351,363,364].

Finally, we should keep in mind that virology has greatly contributed to our current understanding of the nuclear export mechanisms of mRNAs. The discovery of NESs and their importance in CRM1-mediated export and of CTEs and the NXF1–NXT1 export receptor marked a milestone in our understanding of the mechanisms by which mRNAs are recruited for export into the cytoplasm.

Much knowledge has been accumulated on the nuclear export of viral mRNAs, showing that viruses, by evolving and adapting to their hosts, have developed very different strategies to hijack the cellular export machinery and ensure the efficient expression of their own proteins. Some of these strategies, which are not completely understood because they are highly complex and vary according to the species of viral RNA, continue to attract the interest of researchers. Ongoing and future studies should provide a complete picture of the export of mRNAs of viruses, such as hepatitis B virus, influenza viruses, and polyoma- and papillomaviruses. Virology will thus continue to contribute to our understanding of the processes that regulate the post-transcriptional fate of viral and cellular mRNAs.
